# In vivo intratumor angiogenic treatment effects during taxane-based neoadjuvant chemotherapy of ovarian cancer

**DOI:** 10.1186/1471-2407-10-137

**Published:** 2010-04-13

**Authors:** Martin Pölcher, Christian Rudlowski, Nicolaus Friedrichs, Marieke Mielich, Tobias Höller, Mathias Wolfgarten, Kirsten Kübler, Reinhard Büttner, Walther Kuhn, Michael Braun

**Affiliations:** 1 Department of Gynecology and Obstetrics, Center for Integrated Oncology, Bonn University Medical Center, Germany; 2 Institute of Pathology, Center for Integrated Oncology, Bonn University Medical Center, Germany; 3 Institute for Medical Biometry, Informatics, and Epidemiology, University of Bonn, Germany; 4 Zentrale Klinische Forschung, University Freiburg, Germany

## Abstract

**Background:**

The aim of our study was to analyze the effect of taxane-based chemotherapy on tumor angiogenesis in patients with advanced epithelial ovarian cancer.

**Methods:**

Within a prospective phase II trial, 32 patients with stage IIIC and IV ovarian cancer were treated with either two or three cycles of neoadjuvant chemotherapy prior to cytoreductive surgery. Carboplatin (AUC5) and docetaxel (75 mg/m^2^) were administered intravenously in a 3-weekly schedule. Changes in intratumor microvessel density (MVD) were assessed with immunohistochemistry by staining pre- and posttreatment surgical tumor specimens with panendothelial, neovascular and lymphatic vessel markers.

**Results:**

Mean values of MVD defined by CD31, CD34, CD105 and D2-40 antibodies showed 12.3, 21.0, 2.7 and 3.1 vessels per high power field (HPF) before chemotherapy and increased after treatment to 15.3, 21.8, 4.8 and 3.6 per HPF, respectively. These changes were significant for CD31 (p = 0.04) and for CD105 (p = 0.02).

**Conclusion:**

Taxane-based chemotherapy appears to promote tumor vascularization when administered every 3 weeks. A possible explanation is the secondary recovery of MVD in response to immediate cytotoxic and antiangiogenic effects of the chemotherapy. If confirmed prospectively, these findings favor shorter treatment intervals of taxane-based chemotherapy to counteract proangiogenic recovery.

## Background

Although about 80% of advanced ovarian cancer patients respond well to standard management - primary cytoreductive surgery followed by platinum/taxane-based chemotherapy - the majority of patients develop recurrent disease and die of progressive disease[1]. Therefore changes in therapeutic procedures are of clinical interest.

The application of neoadjuvant chemotherapy in advanced ovarian cancer is the subject of past and recent study efforts [2-4]. Results of a recently reported phase 3 study with 704 patients enrolled and a treatment schedule with 3 cycles carboplatin/paclitaxel preoperatively demonstrated that neoadjuvant chemotherapy produces similar PFS and OS rates compared to standard primary cytoreductive surgery and provides a significantly lower perioperative morbidity and mortality in the group treated with neoadjuvant chemotherapy [5].

Preoperative chemotherapy provides an excellent opportunity to analyze cytotoxic therapy effects on the tumor microenvironment; an area of research which received little consideration in the literature so far. Malignant tumors are regarded as complex tissues in which genetically altered malignant cells interact with several normal cell types that collaborate and support malignant growth [6] Based on the impact of neovascularization which contributes to the growth of a tumor mass and the formation of metastases, great efforts have been undertaken to develop therapeutic tools to target this process [7].

Intratumor microvessel density (MVD) has been used to examine the role of vascularization within the malignant process. High MVD is associated with parameters of tumor aggressiveness such as greater incidence of metastases and decreased survival [8]. It was found to have independent prognostic significance when compared with traditional prognostic markers by multivariate analysis in many types of cancer. While the prognostic impact of high MVD In ovarian cancer patients was demonstrated in numerous retrospective studies [9-14], other studies failed to prove a significant association [15-17].

CD31, a transmembrane glycoprotein found at the intercellular junctions of endothelial cells and CD34, a surface glycoprotein of unknown function serve as panendothelial markers, whereas CD105 (endoglin) is expressed almost exclusively on proliferating endothelial cells that are induced by tumoral factors for neoangiogenesis [18-20]. D2-40, a monoclonal antibody, specifically recognises podoplanin and is the most sensitive and specific antibody for the detection of lymphatic endothelium [21].

Anticancer chemotherapeutic agents are known to directly inhibit tumor cell proliferation. In addition, chemotherapeutic drugs were reported to have antiangiogenic activity [22].

The aim of our study was to assess the chemotherapeutic effect on angiogenesis by comparing tumor vasculature before and after neoadjuvant chemotherapy in ovarian cancer patients.

## Methods

Between February 2003 through March 2008, patients with advanced stage IIIC and IV ovarian cancer and large volume ascites (>500 mL) were treated with neoadjuvant chemotherapy as part of a multicenter prospective randomized phase 2 trial [23]. This trial was planned to evaluate response to neoadjuvant chemotherapy, and to analyze surgical outcome. Treatment consisted of either two or three of six cycles of intravenous carboplatin (area under the curve 5) and 75 mg/m^2 ^of docetaxel at 21-day intervals before cytoreductive surgery in order to find a suitable regimen for a planned phase 3 trial. All patients were regularly followed up at 3-month intervals for the first two years and at 6-month intervals thereafter.

To confirm the diagnosis laparoscopic biopsy was performed before chemotherapy and tumor samples were taken from the ovary and/or the peritoneum of the abdominal wall. Cytoreductive surgery was performed within four weeks of the last scheduled chemotherapy cycle and tumor tissue was excised at the beginning of cytoreductive surgical procedures. If no macroscopic residual tumor was detectable at the ovary and/or the peritoneum of the abdominal wall, any macroscopic tumor tissue was obtained irrespective of the anatomic site.

This is a single institution analysis. Of 93 patients enrolled in the multicenter study, 43 patients were treated at the University of Bonn Medical Center and form the basis of our analysis. Tissue sampling and documentation was performed in a standardized fashion. Of the 43 patients, in 32 cases, paired tissue samples from laparoscopy and laparotomy were assessable for the evaluation of MVD before and after treatment.

None of the patients received erythropoetin stimulating agents before cytoreductive surgery. The protocol was approved by the institutional review board. All patients gave written informed consent.

### Immunohistochemistry

Original hematoxylin and eosin stained slides were reviewed by a board certified pathologist (N.F.). Corresponding tumor blocks were obtained. Formalin-fixed tissue specimens were embedded in paraffin and 2 μm sections were dried overnight at room temperature, deparaffinized and rehydrated by decreasing concentrations of ethanol followed by incubation in Tris buffer. For antigen retrieval, the sections were microwaved twice for 15 min at 600 W in 10 mM citrate buffer, pH 6. The following steps were performed semiautomatically using a streptavidin-biotin-peroxidase technique and a DAKO TechMate™ 500 following the instructions of the provider (Dako, Hamburg, Germany). Positive control experiments were performed using tissue slides with microvessels and negative controls by substituting the primary antibody by non-specific immunoglobulin. All specimens were stained within the same pass.

The following primary antibodies were used for immunohistochemical detection: anti-human CD 31 (Clone JG 70 A, 1:100; DAKO), anti-human CD34 (Clone QBEnd-10, 1:100; DAKO), anti CD 105 (Clone SN6 h, 1:50; DAKO) and anti-human D2-40 (Clone D2-40, 1:50; Signet, Dedham, MA, USA).

MVD was measured as reported by Weidner et al. [24]. Using a light microscope, an experienced blinded investigator screened with a 40× magnification for a single area of invasive tumor representative of the highest microvessel density (neovascular "hotspot"). Hotspots were defined as the area of greatest vasculature within the tumor epithelium and immediate adjacent stroma. Areas of stroma without tumor cells were not considered. Vessels with muscular walls were excluded. The number of positively stained vessels was counted in 3 high power fields (200× or 0.74 mm^2^) in each tissue sample and the average value was used as a basis for calculating.

### Statistical analysis

Statistical analysis was performed using the software SAS 9.1.3 (SAS Institute Inc. Cary, NC, USA) and included non-parametric group comparison tests (Mann-Whitney and Wilcoxon-test) nonparametric (Spearman) correlation tests, and parametric (Cox proportional) and nonparametric (Kaplan-Meier) survival analysis with p < 0.05 considered significant.

## Results

The clinical and histological characteristics of the patients included in this study are summarized in Table 1. Ascites volume, target lesions and CA 125 serum levels were compared to pretreatment findings before cytoreductive surgery. All patients responded to the initial preoperative treatment.

**Table 1:** Patient characteristics

	N
Number of patients	32

Age -- (y)	
Median	60
Range	34-78

Histological type	
serous/serous-papillary	31
Endometrioid	1

Histological grade	
G 2	12
G 3	20

Stage	
III C	30
IV	2

CA 125 (U/mL)	
Median	1376
Range	(86-9030)

CA 125 (U/mL) after preoperative treatment	
Median	71
Range	(17-2794)

Number of chemotherapy cylcles before cytoreductive surgery	
Two	19
Three	13

Residual disease after cytoreductive surgery	
no gross residual disease	9
≤ 1 cm	19
>1 cm	4

Lymph node status	
N0*	13
N1	7
NX	12

Recurrence status (%)	
recurrent disease	21
Dead	17

*at least 25 lymph nodes

For each marker, pre- and posttreatment specimens of 32 patients were analyzed. In three cases the pretreatment specimens showed severe artefacts, which prevented their use for the designated analyses due to poor quality. Pre- and posttreatment values were excluded pairwise in these cases.

**Figure 1:**
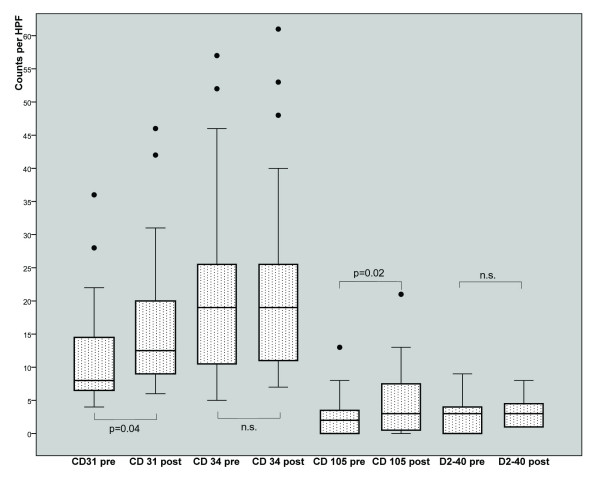
**Mean values of microvessel density per high powerfield in 32 tumor samples before and after neoadjuvant chemotherapy in ovarian cancer patients**.

The highest vessel counts were found by immunostaining with anti-CD34 antibody. There was a strong correlation between both panendothelial markers CD34 and CD31 (Spearman-Rho correlation coefficient 0.67, significance <0.01). By comparison, the selective staining of CD105 (neovascularisation) and D2-40 (lymphatic vessels) generated clearly lower values of MVD (Figure 1).

**Figure 2:**
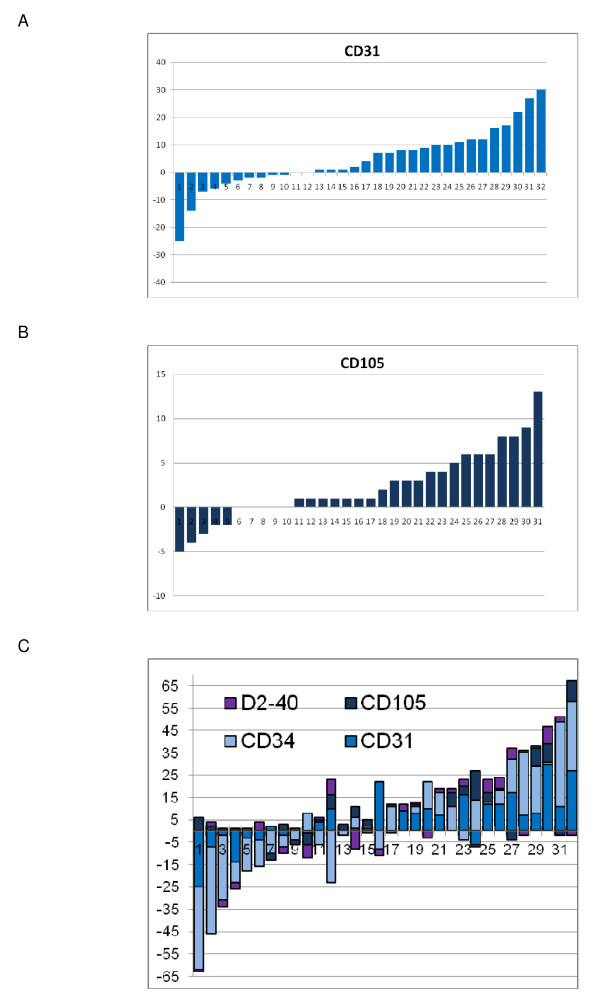
**Changes of microvessel density per high powerfield in 32 tumor samples after chemotherapy treatment**. Waterfall plots for (A) panendothelial (CD31) and (B) selective endothelial markers (CD105); (C) joint illustration of all markers.

Changes from pre- to posttreatment MVD are shown in Figure 2. CD31 and CD105 values showed a significant increase after treatment (p = 0.04 and p = 0.016). There was no significant difference in the mean changes of all MVD markers between two and three cycles of treatment (data not shown). Changes of MVD markers (CD31, CD34 and CD105) were not associated with grade or residual tumor after surgery and further, changes of the lymphatic vessel marker D2-40 were not associated with nodal involvement, grade or residual tumor after surgery.

Mean follow up was 24 months (7 to 65 months), median progression-free survival (PFS) was 13.3 months (95% Confidence Interval (CI) 10.9 to 15.7) and median overall survival (OS) was 36.0 (95% CI 15.9 to 56.0) months. After dichotomization at the median into groups expressing high or low levels of CD31, CD34, CD 105 and D2-40 no significant differences were predictable in the univariate analyses for PFS and OS in both, pretreatment and posttreatment values. The same applies to comparing patients with increasing or decreasing levels of MVD after therapy.

**Figure 3:**
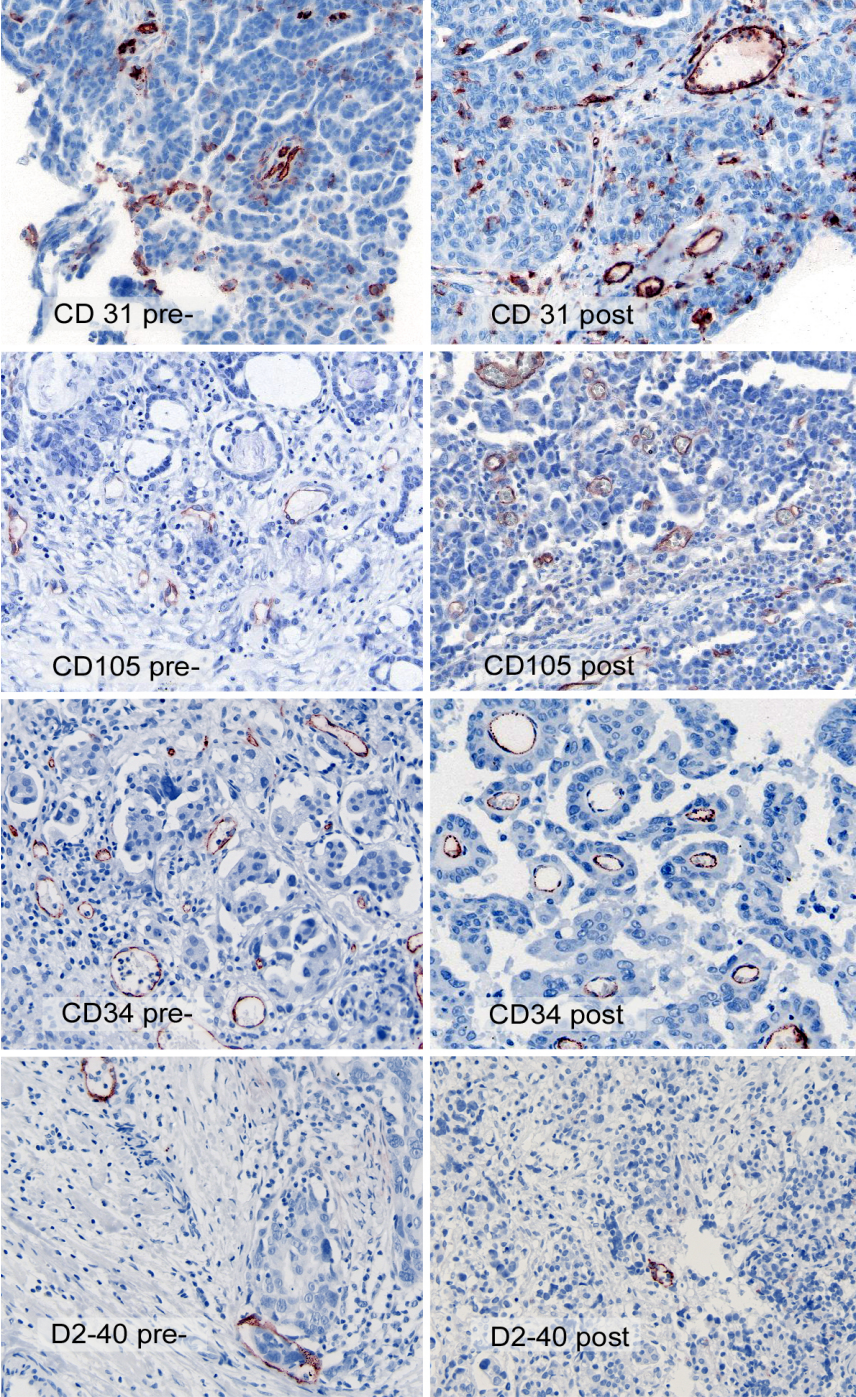
**Representative images of the immunostaining showing antigen expression in pre- and posttreatment samples of patients treated with neoadjuvant chemotherapy**.

Representative images of the microvessel immunostainings are shown in Figure 3.

## Discussion

While chemotherapy has been reported to target endothelial cells in blood capillaries, this study demonstrates that taxane-based neoadjuvant chemotherapy in ovarian cancer does not exert antiangiogenic treatment effects in residual tumor foci.

Although endothelial cells are assumed to be genetically stable and have a low mutation rate, antineoplastic cytotoxic agents are regarded as inhibitors of angiogenesis [25]. Taxanes are microtubule-stabilizing drugs and inhibit endothelial cell proliferation and tubule formation in vitro [26,27]. Docetaxel, which is the study medication of this trial, appears to be more potent at inhibiting angiogenesis in vitro and in vivo than paclitaxel [28], which is the standard medication for treatment of advanced ovarian cancer patients. Furthermore, antiangiogenic activity is not only thought to be mediated by direct effects on endothelial cells but it is also thought to be mediated through effects on cancer cells. Killing cancer cells and eliminating critical cell survival or pro-angiogenic factors (e.g. vascular endothelial growth factor), also affects the endothelial cell compartment and causes antiangiogenic activity [29]. Therefore, comparing tumor samples taken before and after neoadjuvant chemotherapy, one would expect to find a reduction of microvessel density (MVD) in the posttreatment specimens by an inhibition of tumor angiogenesis.

However, in the current analysis all vessel markers showed increasing mean levels after treatment, indicating a lack of a relevant antiangiogenic treatment effect.

CD105 has been considered as a specific and sensitive marker to detect newly sprouting vessels [19]. In two-thirds of the tumor specimens posttreatment samples showed an increased number of CD105 positive vessels as a sign of newly generated vessels. Lymph vessels however, visualized by D2-40 did not show changes in the tumor environment. An inhibitory effect of chemotherapy or, inversely, an increase of the lymph vessel density due to a proangiogenic effect was not found in this study.

Tumor and host-mediated pathways might inverse antiangiogenic treatment effects. There is a growing body of evidence suggesting that circulating bone marrow derived endothelial progenitor cells (CEP) are able to support the vascularization of tumors and may therefore play a synergistic role with angiogenesis [30]. Tumor vasculature does not necessarily derive from endothelial cell sprouting, but CEP home to sites of neovascularization and differentiate into endothelial cells [31]. Recent data demonstrates that some chemotherapeutic drugs can cause simultaneously host-mediated counterregulatory responses from the bone-marrow resulting in tumor angiogenesis and vasculogenesis [32]. This mobilization effect in CEP levels may facilitate tumor cell repopulation during the common time intervals between the individual chemotherapy cycles [33].

Chemotherapy is optimally given at a maximum tolerated dose, with off-therapy intervals of 3 weeks to rescue bone marrow and intestine. Antiangiogenic therapy requires that endothelial cells be exposed to steady blood levels of the inhibitor [34]. Most recent investigations on the antiangiogenic efficacy of different application schedules suggested the use of a tightly spaced, continuous application of appropriate anticancer chemotherapeutic agents [35,36]. These application schedules are able to exert a strong antiangiogenic effect as indicated by an increase of apoptosis of tumor endothelial cells [37]. Furthermore, the mobilization of CEP is decreased in these schedules [33,38]. It has been hypothesized that a more frequent administration of paclitaxel exhibits proapoptotic and antiangiogenic properties and therefore increases its antineoplastic effect [39,40].

A phase 3 trial (JGOG3016) with 637 advanced ovarian cancer patients enrolled compared a dosedense weekly paclitaxel regimen (Carboplatin AUC 6 q21d and Paclitaxel 80 mg/m^2 ^d1, 8, 15, × 6-9) with standard treatment (Carboplatin AUC 6 and Paclitaxel 180 mg/m^2 ^q21 × 6-9) after cytoreductive surgery [41]. This study demonstrated a significant improvement of PFS in the arm with weekly taxane (28 vs.17 months; p = 0.02) and a significant improvement in the overall survival rate after 3-years (72 vs. 65%; p = 0.03). Increased doses of paclitaxel of 200 mg/m^2^, 225 mg/m^2 ^or 250 mg/m^2 ^given every 3 weeks have not shown a benefit in survival rates compared to standard dosage (175 mg/m^2^) [42-44]. The authors concluded that higher survival rates without improved response rates in this study might be attributed to an additional antiangiogenic effect of the weekly application of paclitaxel.

We are aware of several limitations in our experimental approach. Laparoscopic biopsies are mostly smaller than tumor specimens after cytoreductive surgery and may have fewer vascular "hotspots". Therefore initial MVD may be underestimated. We tried to avoid paired sampling from different sites at the pre- and posttreatment surgical procedures, but this was not always feasible due to tumor response after neoadjuvant chemotherapy. However, the immunohistochemistry analysis is based on exclusively pathologically confirmed vital tumor tissue. Therefore, significant differences in MVD derived from different sites are possible but unlikely.

Inflammatory cells, namely monocytes, macrophages, T lymphocytes and neutrophils, fully participate in the angiogenic process by secreting cytokines that may affect endothelial cell functions, including proliferation, migration and activation. Therefore, MVD might also be affected by an immune response to tumor cell damage. The timing of surgery after the preoperative regimen may be crucial, at least with respect to the expression of these MVD markers. All patients included in this study underwent cytoreductive surgery at least after 21 days and within 35 days after the completion of preoperative chemotherapy. The long drug-free periods might provide time for endothelial cells to repopulate the damaged tissues, thereby reducing an antiangiogenic effects of these drugs [45].

Neither pretreatment MVD nor MVD changes after chemotherapy examined here were associated with survival. However, the limited sample size and the inclusion of patients with unfavorable prognostic markers (ascites, stage IIIC and IV) decrease the ability to demonstrate any effect on survival.

Specific antiangiogenic therapy targeting the vascular epithelial growth factor (VEGF) - pathway has found its way into clinical trials and first results show promise for this approach in ovarian cancer treatment [46,47]. Many unanswered questions remain to be clarified. It is not clear whether combination or concurrent antiangiogenic therapy is more sufficient or whether maintenance treatment should be pursued. Recently, converse treatment effects were reported as antiangiogenic therapy elicited malignant progression in animal trials [48,49].

Our data indicates that neoadjuvant chemotherapy provides an excellent opportunity to in vivo assess changes in the tumor environment by comparing pre- and posttreatment samples. This may be helpful to identify treatment effects of cytotoxic drugs.

## Conclusion

These findings suggest that in addition to the cytotoxic effect, the taxane-based chemotherapy cannot exert its antiangiogenic effect within a 3 weekly schedule. A possible explanation is the secondary recovery of MVD in response to immediate cytotoxic and antiangiogenic effects of taxane-based chemotherapy. If confirmed prospectively, these findings favor shorter treatment intervals of taxane-based chemotherapy to counteract proangiogenic recovery.

## Competing interests

Walther Kuhn (Principal Investigator PRIMOVAR trial): Research Funding (Sanofi-Aventis). All other authors indicated no potential conflict of interest.

## Authors' contributions

**MP **designed research, acquired, analyzed data and drafted the manuscript. **CR **provided clinical and scientific support, performed surgical interventions, reviewed the manuscript. **NF **was responsible for the histopathological revision of the patient's samples and for analyzing immunohistochemical staining. **MM **acquisition and analysis of clinical and histopathological data, collected samples, coordinated staining. **TH **performed the statistical analysis. **MW **provided clinical and scientific support, performed surgical interventions, collected follow-up data. **KK **provided clinical and scientific support, applied chemotherapies within the PRIMOVAR trial. **RB**, Head of the Institute for Pathology, reviewed the manuscript. **WK**, Head of the Dept. of Obstetrics and Gynecology, Principle investigator of the PRIMOVAR trial, made a major financial, clinical and critical contribution to this work. **MB **co-designed and supervised the study, assisted in the writing of the manuscript.

All authors read and approved the final manuscript.

Presented in part at the ESGO European Meeting, Belgrade 2009

## Pre-publication history

The pre-publication history for this paper can be accessed here:

http://www.biomedcentral.com/1471-2407/10/137/prepub
